# Zoo professionals and volunteers in the U.S: experiences and prevalence of burnout, mental health, and animal loss

**DOI:** 10.3389/fpsyt.2024.1373525

**Published:** 2024-04-03

**Authors:** Shelby E. McDonald, Lori R. Kogan, Nichole L. Nageotte, Jennifer Currin-McCulloch, Rachel Dickler-Mann

**Affiliations:** ^1^Department of Community Research and Evaluation, Denver Zoological Foundation, Denver, CO, United States; ^2^Clinical Sciences, Colorado State University, Fort Collins, CO, United States; ^3^School of Social Work, Colorado State University, Fort Collins, CO, United States

**Keywords:** zoos, loss, grief, mental health, burnout, resilience, animal care, animal health

## Abstract

**Introduction:**

Burnout and mental health among animal care and health professionals (ACHPs) has received increasing attention in recent years. Despite rapid growth of research in this area, the wellbeing of individuals who work and/or volunteer in zoo settings has received minimal attention.

**Method:**

An anonymous online survey was created to evaluate zoo staff and volunteers’ experiences of animal-related loss, rates of professional fulfillment and burnout, mental health, perceived organizational support, and resilience. Participants included 1695 zoo professionals (72% ACHPs, 20% other staff) and volunteers (7%) who were recruited through relevant professional listservs and online platforms, and flyers on zoo grounds.

**Results:**

ACHPs reported higher levels of anxiety, depression, and burnout and lower levels of professional fulfillment than other zoo staff and volunteers. The most common animal-related losses experienced by ACHPs in the past year were unexpected death (80%) and anticipated loss (74%), with more than half of these losses occurring within the past 3 months. ACHPs’ reported bond with animals under their care was positively associated with depression and anxiety. Having a formal ritual or process following the death of an animal was positively associated with job fulfillment and perceived organizational support and negatively associated with depression and burnout—yet only 17% of participants in our sample indicated that their zoo had such a process or ritual.

**Discussion:**

Our findings suggest that many ACHPs are struggling with burnout, anxiety, depression, and low rates of professional fulfilment and perceived organizational support. We recommend that zoos develop organizational plans that foster a culture which normalizes and validates grief/loss experiences and is proactive in responding to animal loss, related trauma, and other occupational stressors. The results of this research demonstrate the need for systemic changes within the zoo industry, for the betterment and welfare of both humans and the animals under their care.

## 1 Introduction

In recent years, the issue of burnout and mental health among animal care and health professionals (ACHPs) has received increasing attention in the fields of veterinary science and human-animal interaction (1–7). ACHPs is a broad term referring to employees or volunteers who look after animals, with roles including, but not limited to, veterinarians, veterinary nurses, animal attendants, wildlife carers, foster carers, zookeepers, and administrative workers (3). There is growing evidence that ACHPs have higher rates of stress-related mental health problems than the general population (3). When ACHP well-being is not adequately addressed, exposure to occupational stressors (e.g., animal injury, illness, suffering, euthanasia, death) on a routine basis can lead to compassion fatigue, burnout, and mental health issues (5, 8). Despite rapid growth in this area of research, individuals who work and/or volunteer in zoo settings have not been adequately evaluated. The current study addresses this gap by examining experiences of animal-related loss, rates of professional fulfillment and burnout, mental health, perceived organizational support, and resilience in a sample of zoo professionals and volunteers in the United States.

### 1.1 Burnout and mental health

The impacts of occupational stressors such as depression, burnout, and grief and loss, as well as the circumstances contributing to these experiences, are not well understood in the context of contemporary zoos. However, occupational stressors and their impacts are well-documented in similar professions that center on animal care and husbandry, such as general veterinary practice (9–12), animal laboratory settings (13–15), and animal shelters (16–18). Multiple studies have found high rates of PTSD symptoms, psychological distress, burnout, and grief among ACHPs across these settings (3, 6, 16, 19–28). For example, a recent study of animal shelter staff demonstrated that these employees often suffer from secondary traumatic stress (10); moreover, the chance that U.S. animal shelter employees will have posttraumatic stress disorder is five times higher compared to the national average (16). Similarly, studies suggest that veterinary professionals experience high rates of compassion fatigue, secondary traumatic stress, burnout, anxiety, depression, and suicide ideation (6, 23, 29–36).

Occupational stressors that contribute to these outcomes include emotional and moral challenges such as exposure to animal suffering, injury, and death (8). Other stressors include difficult work schedules, financial challenges (educational debt, low-pay), management dissatisfaction, excessive workloads, role ambiguity, physically demanding and exhausting job duties, and the cumulative exposures to highly stressful work events (7, 21, 31, 37). ACHPs and allied professionals in zoo settings experience similar stressors to these populations [e.g., exposure to euthanasia. animal illness, animal transfers; (38, 39)] and like ACHPs in other settings, often become attached to animals in their care (39). Marino (40) examined experiences of burnout in a convenience sample of 616 people who currently or previously worked at zoos and aquariums. This study found that 91% of the sample reported they experienced burnout while working at a zoo or aquarium, and 60% stated they left a position because of burnout. They found that participants who identified as Black, Indigenous, and/or people of color were more likely than those who identified as white to leave their positions because of burnout. Additionally, participants who experienced harassment and discrimination in their workplace were also significantly more likely to experience burnout and leave their positions because of it. Thus, there is an urgent need to better understand rates of burnout and mental health among these professionals. Further, understanding the types of animal losses zoo staff and volunteers experience (e.g., anticipated loss, unexpected loss, animal transfers), and how these types of losses are associated with burnout and wellbeing, could assist the zoo industry in understanding how to best support their staff and volunteers in coping with typical job stressors and guide evidence-based practices to promote mental health and wellbeing in this industry.

### 1.2 Factors that may facilitate wellbeing

In addition to emotional and moral challenges, it is also important to focus research attention on understanding positive aspects of work in zoo settings and to identify how factors such as individual resilience, organizational support, and professional fulfillment are associated with zoo staff and volunteers’ wellbeing and ability to adaptively cope with typical occupational stressors. Personal resilience is a term often used to refer to an individual’s ability to adjust to adversity or setbacks, retain a sense of control over their environment, and continue to persist in a healthy and adaptive manner (41). Resilience is often negatively associated with burnout in health professionals (42, 43), and prior work highlights the importance of an individual’s personal resilience in the context of stressful occupations, including animal care work (44, 45). In addition, organizational support has also been identified as an important factor that may influence the wellbeing of ACHPs and individuals who work in other stressful professions (8, 42, 46–48). Indeed, a recent study of Australian ACHPs found that perceived organizational support accounted for approximately 17% of the variance in burnout (8). Moreover, perceived organizational support in this sample was found to be inversely related to anxiety, depression, PTSD symptoms, grief, and stress. Relatedly, there is some evidence that professional fulfillment may be associated with higher levels of well-being and reduce feelings of burnout among some groups of ACHPs (although emerging evidence suggests low rates of professional fulfillment among some ACHPs [i.e., veterinary technicians (11), shelter veterinarians (49)]). For example, Wallace (50) found that veterinarians in clinical practice who felt their work was fulfilling and meaningful reported higher levels of wellbeing.

### 1.3 Zoo volunteers

There has been minimal research on these topics for zoo professionals but even less for those who volunteer within zoos. Many organizations in the U.S., particularly non-profits, rely heavily on volunteers. This is especially true for zoos across the United States, which frequently rely on volunteers for animal care, education, conservation efforts, and other programming (51, 52). Zoo volunteers may experience similar stressors and outcomes as paid employees; moreover, factors that support their wellbeing may be comparable to or different from paid staff (52). Research on volunteers in other non-profit settings centered on animal care and rehabilitation suggests that volunteers experience compassion fatigue (18, 53). Additionally, there is some evidence that rates of compassion fatigue (comprising burnout and secondary traumatic stress) are comparable between paid staff and volunteers in animal shelter settings (54). Given links between stress and turnover [e.g., 25, 55)], it is important for the zoo industry to invest in identifying factors associated with zoo volunteers’ wellbeing.

### 1.4 Current study

Understanding rates and types of animal loss, burnout, and mental health among zoo professionals and volunteers can help guide the zoo industry in efforts to better support and care for the individuals who carry out their mission. Additionally, understanding zoo professionals’ perceptions of professional fulfillment, organizational support, and personal resilience has implications for informing strategies to support positive coping and wellbeing among people who work and volunteer in this industry. To this end, the current study was designed to survey current zoo professionals and volunteers working at AZA (Association of Zoos and Aquariums) accredited institutions within the United States. Specifically, we aimed to examine rates of animal loss, professional fulfillment, burnout, anxiety, depression, resiliency and perceived organizational support among zoo professionals and volunteers, and differences in these rates between ACHPs, other zoo staff, and volunteers. We also aimed to identify predictors of job fulfillment, burnout, depression, anxiety, and perceived organizational support among ACHPs, adjusting for the potential confounding effects of demographic factors. This study was exploratory and, therefore, there were no specific hypotheses.

## 2 Materials and method

### 2.1 Study design

An anonymous online survey was created to evaluate zoo staff and volunteers’ experiences of animal-related loss and grief in the workplace. Related constructs including professional fulfillment and burnout, organizational support, anxiety and depression, and resilience were also assessed. The survey was created and tested by researchers at Denver Zoological Foundation (Denver Zoo) and Colorado State University after seeking input from several members of the Denver Zoo community. Recruitment and data collection took place from July 26, 2023, through October 15, 2023. Surveys were completed electronically using the Alchemer survey platform and took approximately 15 minutes to complete. The survey was only offered in English. Following completion of the survey, participants had the option of clicking on an external link to an electronic form where they could provide their name and contact information for the chance to enter a drawing for a catered lunch for themselves and their colleagues ($250). This data was stored separately from the survey data.

We used multiple platforms to recruit our participants. First, information about the study (including the survey link and study flyer) was posted to the AZA website on the AZA network. Specifically, we posted to the following forums: Animal Ambassadors, Continuous Improvement in Zoos and Aquariums, Curators, Education, Research and Technology, Social Science Research and Evaluation Scientific Advisory Group, Volunteer and Intern Engagement, and Volunteer/Docents. An executive team member at Denver Zoo posted the same information to the following AZA groups: Amphibians, Animal Health, Animal Management, Animal Welfare, Avian Interest Group, Chelonians, Crocodilians, General Curators, Lizards, Snakes, and Ungulates. Messages posted to these forums provided the survey link and study flyer and invited forum participants to: a) participate in the survey and/or b) contact the first author if they were interested in sharing this study information with staff, volunteers, and/or their institutional leadership.

Following these recruitment efforts, staff at other zoos (see acknowledgements section) contacted the first author and shared the survey with members of their staff. In addition to the AZA network, we recruited participants through several listservs (e.g., American Association of Zookeepers) and other zoo-related social media pages on Facebook, Instagram and LinkedIn (The Zoo Scientist, Growing Resilience in Zoo and Aquarium Professionals; Association of Minority Zoo & Aquarium Professionals). Study flyers were also posted at the 2023 Annual AZA Conference.

### 2.2 Participants

A total of 2,492 respondents completed the survey screening questions. Participants were eligible to participate in the study if they were currently working or volunteering (and had for at least 6 months) at an AZA accredited zoo within the U.S. A total of 329 responses were disqualified through the screening process and 468 responses were disqualified because they provided only partial responses, leaving a final sample size of 1695 for analysis. The average age of participants in the sample was 37 years (*SD*= 11.6). Study participants predominately identified as female, feminine, or woman (79%) and white (91%) with a Bachelor’s degree (1115, 67%). Twenty percent (N = 334) identified as LGBTQ+, with a majority of these individuals identifying as Bisexual (144, 43%) or Queer (62, 19%). Participant demographics are provided in **Tables 1**–**3**.

**Table 1:** Participants’ reported work or volunteer hours and length of time working in a zoo setting.

Role	ACHP (n = 1252)	Other (n= 332)	Volunteer (n = 111)
Work Schedule	N (%)	N (%)	N (%)
Full Time	1195 (95)	274 (83)	--
Part Time/Variable Part-Time	50 (4)	47 (14)	--
Seasonal	7 (1)	11 (3)	--
Hours volunteered per month	--	--	17.1 (SD = 13.7)
Years working in zoo setting	N (%)	N (%)	N (%)
Less than one year	5 (<1)	13 (4)	3 (3)
1-4 years	174 (14)	100 (30)	29 (26)
5-9 years	359 (29)	101 (30)	27 (24)
10-19 years	428 (34)	77 (23)	32 (29)
20+ years	286 (23)	41 (12)	20 (18)

**Table 2:** Participants’ reported demographics.

Role	ACHP (n = 1252)	Other (n= 332)	Volunteer (n = 111)
Age	35.6 (SD = 9.3)	36.9 (SD = 11.0)	57.0 (SD = 17.9)
Education level	N (%)	N (%)	N (%)
Less than High School	1 (<1)	0	3 (3)
High School or GED	38 (3)	26 (8)	6 (5)
Associate’s degree (2 year)	111 (9)	33 (10)	13 (12)
Bachelor's degree (4 year)	906 (72)	170 (51)	39 (35)
Master's degree	123 (10)	88 (27)	42 (38)
Doctoral degree	52 (4)	9 (3)	6 (5)
Prefer not to say	21 (2)	6 (2)	2 (2)
Ethnicity	N (%)	N (%)	N (%)
Hispanic or Latino	75 (6)	23 (7)	2 (2)
Not Hispanic or Latino	1115 (89)	285 (86)	94 (85)
Prefer not to say	62 (5)	24 (7)	15 (14)
Race*	N (%)	N (%)	N (%)
American Indian or Alaska Native	16 (1)	9 (3)	2 (2)
Asian	27 (2)	9 (3)	1 (1)
Black or African American	24 (2)	13 (4)	0
Native Hawaiian or Other Pacific Islander	8 (1)	1 (<1)	0
White	1155 (92)	285 (86)	98 (88)
Prefer to self-describe	14 (1)	8 (2)	2 (2)
Prefer not to say	55 (4)	25 (8)	12 (11)
Gender identity*	N (%)	N (%)	N (%)
Agender	3 (<1)	1 (<1)	0
Female, feminine, or woman	1006 (80)	236 (71)	91 (82)
Genderfluid	3 (<1)	3 (1)	1 (1)
Genderqueer or non-binary	20 (2)	10 (3)	0
Gender non-conforming	4 (<1)	2 (1)	0
Intersex	0	0	0
Male, Masculine, or Man	179 (14)	66 (20)	12 (11)
Not cisgender, but I don’t identify with a specific identify	9 (1)	0	0
Questioning or figuring it out	4 (<1)	2 (1)	0
Transgender	6 (1)	2 (1)	0
Two-spirit or other Traditional or Indigenous genders	1 (<1)	0	0
Prefer not to respond	35 (3)	15 (5)	5 (5)
I don’t understand the question	5 (<1)	3 (1)	2 (2)
Prefer to self-identify	1 (<1)	0	0

* Participants could select more than one response.

**Table 3:** Participants’ reported identification as LGBTQ+ and sexual orientation of those who identified as LGBTQ+.

Role	ACHP (n = 1252)	Other (n= 332)	Volunteer (n = 111)
Identify as LGBTQ+*	N (%)	N (%)	N (%)
Yes	249 (20)	76 (23)	9 (8)
No	916 (73)	225 (68)	93 (84)
Unsure	34 (3)	12 (4)	2 (2)
Prefer not to say	53 (4)	19 (6)	7 (6)
Sexual orientation*	(n=249)	(N = 76)	N = 9
Asexual or Ace spectrum	29 (12)	11 (15)	0
Bisexual	106 (43)	34 (45)	4 (44)
Gay	37 (15)	15 (20)	0
Lesbian	42 (17)	12 (16)	4 (44)
Not heterosexual but don’t identify with a specific identity	3 (1)	1 (1)	0
Pansexual or Omnisexual	31 (12)	11 (15)	1 (11)
Questioning or figuring it out	5 (2)	2 (3)	0
Straight or heterosexual	3 (1)	0	0
Queer	40 (16)	22 (29)	0
Prefer not to respond	7 (3)	0	0
I don’t understand the question	0	0	0
Prefer to self-identify	2 (1)	1 (1)	0

*Participants could select more than one response.

### 2.3 Measures

#### 2.3.1 Professional fulfillment and burnout

The Stanford Professional Fulfillment Index [PFI; (56)] was used to assess self-reported professional fulfillment and professional burnout. The PFI includes a 6-item Professional Fulfillment subscale (e.g., “I feel happy at the zoo”; “I feel in control when dealing with difficult problems at the zoo”), a 6-item Interpersonal Disengagement subscale (e.g., “Less empathetic with my colleagues”; “Less connected with zoo animals”), and a 4-item Work Exhaustion subscale (e.g., “A sense of dread when I think about the work I have to do”; “Lacking in enthusiasm at the zoo”). For all items, participants were instructed to reflect on their past two weeks and indicate how well the items described their experience using a 5-point scale ranging from 1 (not at all) to 5 (extremely). An average score was created for the Professional Fulfillment sub-scale; the initial validation study of the PFI reported evidence of the utility of a cut-point (cut point = 3.0) to identify dichotomous groupings that distinguish participants who were experiencing professional fulfillment and those who were not. Scores for the Work Exhaustion and Interpersonal Disengagement sub-scales were combined to assess burnout (score 0-10) with higher scores indicating more burnout symptoms. A cut-point of 1.33 was used to identify dichotomous groupings that distinguished participants experiencing burnout from those who were not. Prior studies indicate the PFI is a valid and reliable assessment of professional fulfillment and burnout (11, 56). In the current sample, reliability of the professional fulfillment and burnout scales were excellent (McDonald’s omega= 0.895 and 0.910, respectively; Cronbach’s alpha= 0.894 and 0.909, respectively).

#### 2.3.2 Perceived organizational support

A modified, 5-item version of the Perceived Organizational Support Scale [POS; (57)] was used to gather staff and volunteer perceptions of the degree to which their organization valued their contributions, and actions the organization might take that could affect the wellbeing of the employee. Responses were measured on a 7-point Likert type scale (1-strongly disagree to 7- strongly agree). Items were adapted so that the word “organization” was replaced with the word “zoo” (i.e., “The zoo values my contribution to its well-being”; “If the zoo could hire someone to replace me at a lower salary it would do so” [reverse scored]; “The zoo fails to appreciate any extra effort from me [reverse scored]; “The zoo strongly considers my goals and values”; “The zoo would ignore any complaint from me [reverse scored]”). Reverse scored items were recoded so that a high score would indicate a higher degree of POS; the total score was obtained by totaling the 5 items (possible range 7-35). Prior research indicates the POS demonstrates adequate reliability and criterion validity across samples (58, 59). Due to the unidimensional structure and high internal reliability of the scale, prior research indicates shorter versions of the scales do not appear to be problematic (58). Reliability of the adapted 5-item scale utilized in the current study was excellent (McDonald’s omega = 0.880; Cronbach’s alpha = 0.880).

#### 2.3.3 Anxiety and depression

The Patient Health Questionnaire for Depression and Anxiety (PHQ-4), a brief, 4-item self-report measure used to screen for depression and anxiety (60, 61), was used to assess anxiety and depression. The PHQ-4 was developed as a brief tool to identify the severity and frequency of anxiety and depression in community samples. The two-item Anxiety scale prompts participants to evaluate their experiences related to “feeling nervous, anxious or on edge” and “not being able to stop worrying” (possible range: 0-8); The 2-item Depression scale prompts participants to evaluate their experiences related to “feeling down, depressed or hopeless” and “little interest or pleasure in doing things” (possible range 0-8). In the study, we used cut-off scores of 3 to indicate “yellow flags” and scores of 5 or greater as “red flags” for the presence of depression and/or anxiety (61). Prior research indicates the English language version of the PHQ demonstrates high reliability and validity across samples and population groups with varying social locations and cultures (61–65). Prior studies also indicate this tool is a reliable and valid instrument for screening anxiety and depression in both clinical and non-clinical populations (66). Reliability estimates in the current sample for depression (Cronbach’s alpha = 0.866) and anxiety (Cronbach’s alpha = 0.868) were excellent.

#### 2.3.4 Resilience

We used the Brief Resilience Scale [BRS; (67)] to gain insights into the extent to which zoo staff and volunteers perceive themselves as resilient (having the ability to recover from stress or “bounce back” from adverse events and contexts). The BRS is comprised of six items (e.g., “I tend to bounce back quickly after hard times”; “It does not take me long to recover from a stressful event”). Participants rate the items on a five-point Likert scale (1 = strongly disagree to5 = strongly agree). Three items are reverse coded so that for the total score, a higher score indicates a greater sense of perceived resilience. Prior studies have established the following score interpretation ranges and cutoffs: Low Resilience= 1.00–2.99; Normal Resilience= 3.00–4.30; High Resilience= 4.31–5.00. Previous research indicates that the BRS is reliable and demonstrates evidence of construct validity across diverse samples (68). In the current study, reliability was excellent (McDonald’s omega = 0.869; Cronbach’s alpha= 0.869).

#### 2.3.5 Types of animal-related loss

Participants were provided with a list of animal-related losses and asked to select all that they had experienced in the past 12 months. Specifically, the question read: “There are several types of loss that can occur when working or volunteering within a zoo setting. Which of the following animal-related losses have you experienced in the past 12 months? Select all that apply.” The options included: 1) Anticipated death of an animal I worked closely with, 2) Unexpected death of an animal I worked closely with, 3) Transfer of an animal I worked closely with to a different zoo, and 4) Change in job that led to no longer working with a particular animal. Participants also had the option of selecting “I have not experienced animal-related losses in the past 12 months.” For each response selected, participants were asked a series of follow up questions to understand a) when the loss occurred (e.g., “You indicated that you experienced the anticipated death of an animal you worked closely with. Think about your most recent loss. When did that anticipated loss occur?”) and b) how bonded they were with the animal they lost (i.e., “How bonded were you with that animal”)?. Participants’ level of bond with the animal they lost was measured on a 10-point slider scale, from “Not bonded at all” to “Very bonded.” Response options for the question regarding the timing of the loss were as follows: in the past month, 2-3 months ago, 4-6 months ago, 7-8 months ago, and 9-12 months ago.

Participants who indicated that they had *not* experienced animal-related loss in the past 12 months were then asked if they had *ever* experienced any animal-related losses while working at their current zoological institution. Response options for this question were identical to those in the question about their experiences in the past 12 months; however, their level of bond and the timing of the loss were not assessed.

#### 2.3.6 Demographic questions

Demographic questions were asked at the end of the survey and included questions about participants’ age, gender identity, sexual orientation, race, ethnicity, and education.

### 2.4 Data analysis

After downloading the data from the Alchemer survey platform, descriptive statistics, Analyses of Variance tests, Chi-Square tests, and multiple linear regression were conducted with IBM SPSS Version 26 (IBM, Armonk, NY, USA). Descriptive statistics were calculated to characterize participant demographics. Analyses of variance tests and Chi Square tests were used to assess for differences between the three groups of participants (Animal Care/Health Professional, Other, and Volunteer). We performed a series of multiple linear regression analyses to determine predictive variables for Job Fulfillment, Burnout, Depression, Anxiety, and POS. The potential predictor variables included Animal Loss within past month (yes/no), Bond score, Resiliency score, Ritual following death (yes/no), Age (29 and younger, 30-39 years of age, 40-49 years of age, 50 and older), Length of time working in the field (11 months or less, 1-4 years, 5-9 years, 10-19 years, 20 or more years) and Identification as LGBTQ+ (yes/no). All variables were entered into the models simultaneously. Statistical significance level was set at *p* = 0.05.

## 3 Results

Participants were asked to indicate from a list of 19 options (including ‘Other’) their role at the zoo. The most common roles included Animal care specialist (*n* = 923, 55%), Other (*n* = 184, 11%), Volunteer (*n* = 110, 7%), and Curator (*n* = 105, 6%). Because many of the identified roles contained very small numbers, to ensure anonymity, all paid employees’ responses, including each response under ‘Other’, were recoded into ACHP Professional or Other, resulting in three groupings (“Animal Care/Health Professional” (*n* = 1252, 74%), “Other” (332, 20%) and “Volunteer” (111, 7%)). The majority of employees reported working full-time while volunteers reported volunteering an average of 17 hours per week (**Table 1**).

Participants were asked to indicate, from a series of possible 15 options (including ‘Other’ and ‘Not applicable’), the animals they primarily work with, with the ability to select more than one type. The most common responses for ACHPs were Birds (656, 52%) and Small Mammals (540, 43%). The most common animal responses for other staff were Ambassador animals (109, 33%) and Reptiles (61, 18%), while volunteers reported working most often with Primates (23, 21%), Birds (18, 16%) and Hoofstock (18, 16%) (**Table 4**).

**Table 4:** Primary animal worked with as reported by participants, divided by role.

Role*	ACHP (n = 1252)		Other (n= 332)	Volunteer (n = 111)
	N (%)	Mean bond score (1-10) and SD	N (%)	N (%)
Ambassador animals	382 (31)	5.94 (2.15)	109 (33)	17 (15)
Amphibians	304 (24)	5.45 (2.25)	54 (16)	4 (4)
Birds	656 (52)	6.10 (2.11)	58 (18)	18 (16)
Carnivores	598 (48)	6.26 (2.18)	37 (11)	14 (13)
Domestic animals	259 (21)	5.91 (2.12)	44 (13)	4 (4)
Elephants/pachyderms	197 (16)	5.97 (2.28)	17 (5)	13 (12)
Fish	214 (17)	5.30 (2.19)	19 (6)	3 (3)
Hoofstock	507 (41)	6.18 (2.06)	37 (11)	18 (16)
Invertebrates	254 (20)	5.52 (2.25)	46 (14)	5 (5)
Marine mammals	158 (13)	5.76 (2.26)	14 (4)	1 (1)
Primates	438 (35)	6.15 (2.26)	28 (8)	23 (21)
Reptiles	454 (36)	5.88 (2.26)	61 (18)	8 (7)
Small mammals	540 (43)	6.22 (2.26)	55 (16)	16 (14)
Other	36 (3)	--	19 (6)	9 (8)
Not applicable	20 (2)	--	166 (50)	38 (34)

*Participants could select more than one response.

### 3.1 The Stanford Professional Fulfillment Index

The mean of all participants’ Professional Fulfillment PFI score was 2.44 (*SD* = 0.87). Using the cut-off point of 3.0 or higher, 31% of the total sample reported experiencing professional fulfillment. Analysis of Variance was used to explore differences in fulfillment level between each of the three roles: ACHPs, Other, and Volunteer. There was a significant difference between each group (*F* = 73.43, *p*<.001), with Volunteers reporting the highest rate of Professional Fulfillment (*X=* 3.18, *SD* = 0.67), followed by Other (*X=* 2.68, *SD* = 0.86) and then ACHPs (*X=* 2.31, *SD* = 0.84). A total of 76 (68%) Volunteers met or exceeded the cut-off for Professional Fulfillment, compared to 140 (42%) Others and 307 (25%) ACHPs.

### 3.2 Stanford Professional Burnout Index

The overall mean for all participants for the 10-item PFI Burnout scale was 1.34 (*SD* = 0.83). Using Analysis of Variance, a significant difference was found between the ACHPs, Other, and Volunteer groups (*F* = 146.08, *p* <.0001), with ACHPs reporting the highest rates of Burnout (*X=* 1.51, *SD* = 0.79), followed by Other (*X=* 1.04, *SD* = 0.79) and Volunteers (*X=* 0.35, *SD* = 0.45). Based on the cut-point of 1.33 used to identify participants experiencing burnout, a total of 693 (55%) ACHPs were at or above the threshold, compared to 105 (32%) Others and 5 (5%) Volunteers.

### 3.3 Perceived Organizational Support Scale

The mean for the sum of the POS Scale for all participants was 20.51 (*SD* = 7.76). There was a significant difference, based on Analysis of Variance results, between each group (*F* = 73.89, *p* <.0001), with Volunteers scoring the highest (*X=* 27.95, *SD* = 7.76), followed by Other (*X=* 22.78, *SD* = 7.13) and ACHPs (*X=* 19.33, *SD* = 7.65).

### 3.4 The Patient Health Questionnaire - depression

The mean sum of the two items from the PHQ-4 that measure depression was 1.79 (*SD* = 1.81). Using the cutoff value of 3 or above to identify potential depression, 486 (29%) participants met or exceeded this cutoff. There was a significant difference, based on Analysis of Variance results, between all three groups (*F* = 45.42, *p*<.001), with ACHPs scoring the highest (*X=* 1.99, *SD* = 1.83), followed by Others (*X=*1.48, *SD* = 1.72) and Volunteers (*X=* 0.45, *SD* = 0.98). A total of 407 (33%) ACHPs met or exceeded the cutoff for depression. This number was 74 (22%) for Others and 4 (4%) for Volunteers.

### 3.5 The Patient Health Questionnaire – anxiety

The mean sum of the two items from the PHQ-4 that measure anxiety was 2.30 (*SD* = 1.88). Using the cut off value of 3 or above to identify potential anxiety, 645 (38%) met the threshold for anxiety. Using Analysis of Variance, a significant difference was found between all three groups (*F* = 68.21, *p*<.001), with ACHPs scoring the highest (*X=* 2.54, *SD* = 1.87), followed by Others (*X* = 1.99, *SD* = 1.77) and Volunteers (*X =* 0.54, *SD* = 1.09). A total of 532 (43%) ACHPs, 107 (32%) Others, and 5 (5%) Volunteers met or exceeded the cutoff for moderate to severe anxiety.

### 3.6 Brief Resilience Scale

The mean for the Brief Resilience Scale for all participants was 2.54 (*SD* = 1.09). Using the cutoff values of Low (1.00 – 2.99), Normal (3.00 – 4.30) and High (4.31 – 5.00), 402 (24%) participants had scores indicating low resilience, 1123 (66%) had scores indicating normal resilience, and 170 (10%) had scores indicating high resilience. Analysis of Variance results found a significant difference (F = 3.37, p =.034) between ACHPs (*X=* 3.41, SD = 0.73) and Volunteers (*X=* 3.59, *SD* = 0.75). There were no differences between Others (*X=* 3.41, *SD* = 0.70) and ACHPs or Volunteers. The number of ACHPs whose scores suggested high resilience was 124 (10%), compared to 27 (8%) of Others and 19 (17%) of Volunteers.

### 3.7 Types of animal-related loss

For ACHPs, the most common losses in the past 12 months included Unexpected death, experienced by 998 (80%), and Anticipated loss, experienced by 921 (74%). Over half of the ACHPs reported these losses had occurred either within the past month or within the last 2-3 months. Reported mean bond level for all four types of losses ranged from 8.15 (*SD* = 2.22) for Change in Job, to 6.06 (*SD* = 2.74) for Unexpected Death. If participants had not experienced any of the four types of loss within the past 12 months, they were asked if they had ever experienced it. Approximately 80% of these participants reported having experienced Unexpected death or Anticipated death at some point, while 48% reported having experienced a Transfer or a Change in job (**Table 5**). Anticipated death and Unexpected death were also the most commonly reported types of loss for “Other” participants and Volunteers (**Tables 6**, **7**).

**Table 5:** Animal loss: Type, time and bond for ACHP (n = 1252).

Loss in past 12 months	Bond		Time					
			In past month	2-3 months	4-6 months	7-8 months	9-12 months	Ever experienced (but not in past 12 months) n = 80)
			N (%)	N (%)	N (%)	N (%)	N (%)	N (%)
Anticipated death	M 6.09 (SD 2.57)	In past 12 months n = 921	243 (27)	241 (27)	183 (21)	87 (10)	138 (16)	62 (78)
Unexpected death	6.06 (SD 2.74)	In past 12 months n = 998	340 (35)	262 (27)	157 (16)	86 (9)	123 (13)	65 (81)
Transfer	6.52 (SD 2.69)	In past 12 months n = 583	123 (21)	150 (26)	154 (26)	63 (11)	93 (16)	38 (48)
Change in job	8.15 (SD 2.22)	In past 12 months n = 247	42 (17)	50 (20)	54 (22)	32 (13)	69 (28)	38 (48)

**Table 6:** Animal loss: Type, time and bond for Other (n = 332).

Loss in past 12 months	Bond		Time					
			In past month	2-3 months	4-6 months	7-8 months	9-12 months	Ever experienced (but not in past 12 months) n = 131)
			N (%)	N (%)	N (%)	N (%)	N (%)	N (%)
Anticipated death	M 4.86 (SD 2.31)	In past 12 months n = 119	30 (25)	40 (34)	27 (23)	9 (8)	13 (11)	18 (14)
Unexpected death	5.03 (SD 2.72)	In past 12 months n = 160	50 (31)	41 (26)	38 (24)	14 (9)	17 (11)	28 (21)
Transfer	5.28 (SD 2.82)	In past 12 months n = 61	19 (31)	15 (25)	16 (26)	5 (8)	6 (10)	15 (12)
Change in job	7.24 (SD 2.49)	In past 12 months n = 33	2 (6)	9 (27)	11 (33)	3 (9)	8 (24)	13 (10)

**Table 7:** Animal loss: Type, time and bond for Volunteers (n=111).

Loss in past 12 months	Bond		Time					
			In past month	2-3 months	4-6 months	7-8 months	9-12 months	Ever experienced (but not in past 12 months) n = 35)
			N (%)	N (%)	N (%)	N (%)	N (%)	N (%)
Anticipated death	M 4.81 (SD 2.10)	In past 12 months n = 34	2 (6)	13 (38)	11 (32)	2 (6)	6 (18)	4 (11)
Unexpected death	5.29 (SD 2.64)	In past 12 months n = 54	17 (32)	10 (19)	14 (26)	6 (11)	7 (13)	10 (29)
Transfer	6.00 (SD 2.59)	In past 12 months n = 29	5 (17)	7 (24)	6 (21)	4 (14)	7 (24)	9 (26)
Change in job	4.63 (SD 0.74)	In past 12 months n = 8	–	–	1 (13)	1 (13)	6 (75)	3 (9)

A larger percentage of Others and Volunteers, compared to ACHPs, reported not having experienced these types of losses within the past 12 months (Anticipated loss [*X*^2^ = 201.62, *p*<.001]; Unexpected loss [*X*^2^ = 140.42, p<.001], Transfer [*X*^2^ = 102.56, p<.001]; and Change in job [*X^2^ =*30.73, *p*<.001]). Similarly, Others and Volunteers reported they had never experienced any of these types of losses more often than ACHPs (Anticipated loss [*X*^2^ = 99.16, p<.001]; Unexpected loss [*X*^2^ = 76.13, *p*<.001], Transfer [*X*^2^ = 34.25, *p*<.001]; Change in job (*X*^2^ = 45.20, *p*<.001]) (**Tables 5**–**7**). For further analysis, these four types of losses were combined to create one variable that denoted any animal loss. The amount of time since each type of loss was also combined and recoded as a binary variable (within the past month (yes/no). A total of 289 (17%) participants said their zoo had any formal process or ritual that was performed following the death of an animal, 1051 (62%) said no and 355 (21%) reported they did not know.

Because a substantial number of Others (166, 50%) and Volunteers (38, 34%) reported that they do not work directly with animals, the decision was made to analyze the potential predictive value of animal loss (in addition to job and personal factors), on Job Fulfillment, Burnout, Depression, Anxiety, and POS for ACHPs only.

### 3.8 Multiple linear regression analyses

#### 3.8.1 Job fulfillment

The multiple linear regression predicting job fulfillment was significant (*F*(12) = 12.33, *p* < 0.001), with an *R^2^* of 0.134. Significant predictors of Job Fulfillment included Ritual (B = 0.251; *p* < 0.001; higher Job Fulfillment reported by those having a ritual) and Resilience (B = 0.351, p <.001; higher Job Fulfillment reported by those with higher Resilience scores) (**Table 8**).

**Table 8:** Results of the multiple linear regression model predicting Job Fulfillment as a function of animal loss within past month, bond score, resiliency score, formal process/ritual following death, age, length of time working in the field, and identification as LGBTQ+.

ANOVA						
Model	Sum of Squares	df	Mean Squares	F	Sig.	
Regression Total	90.255 5826.42	12 966	7.52	12.33	<0.001	
Coefficients (Dependent Variable: Job Fulfillment)					95.0% CI	
Variable	Coefficient (B)	Std. Error	t	Sig.	Lower Bound	Upper Bound
(Constant)	1.189	.172	6.922	<.001	.852	1.526
Ritual - yes	.251	.065	3.832	<.001	.122	.379
Ritual - no	0	.	.	.	.	.
Bond	.001	.012	.113	.910	-.023	.025
Loss in past month – yes	-.082	.051	-1.602	.109	-.182	.018
Loss in past month - no	0	.	.	.	.	.
Resilience	.351	.035	10.061	<.001	.283	.420
Age 29 and younger	-.172	.120	-1.431	.153	-.408	.064
Age 30-39	-.155	.108	-1.437	.151	-.366	.057
Age 40-49	.008	.105	.072	.943	-.198	.213
Age 50 and older	0	.	.	.	.	.
Time in field – 11 month/less	.177	.148	1.200	.230	-.113	.467
Time in field – 1-4 years	.087	.112	.774	.439	-.134	.308
Time in field – 5-9 years	.037	.110	.333	.740	-.180	.253
Time in field – 10-19 years	-.069	.102	-.671	.502	-.269	.132
Time in field – 20+	0	.	.	.	.	.
LGBTQ+ yes	-.048	.064	-.760	.447	-.173	.077
LGBTQ+ no	0	.	.	.	.	.

#### 3.8.2 Burnout

The multiple linear regression predicting burnout was significant (*F*_(12)_ = 14.49, *p* < 0.001, *R^2^* = 0.154). Significant predictors of burnout included Ritual (B = -0.191; *p* = 0.002; higher burnout reported by those with no ritual), Resilience (B = -0.354, *p* <.001; higher burnout reported by those with lower Resilience), and age (B = 0.336, 0.282 *p* = .004; higher burnout reported by those ages 30-39 and 29 years of age and younger compared to participants 40 years of age or older) (**Table 9**).

**Table 9:** Results of the multiple linear regression model predicting Burnout as a function of animal loss within past month, bond score, resiliency score, formal process/ritual following death, age, length of time working in the field, and identification as LGBTQ+.

ANOVA						
Model	Sum of Squares	df	Mean Squares	F	Sig.	
Regression Total	89.79 2843.90	12 966	7.48	14.49	<0.001	
Coefficients (Dependent Variable: Burnout)					95.0% CI	
Variable	Coefficient (B)	Std. Error	t	Sig.	Lower Bound	Upper Bound
(Constant)	2.629	.158	16.635	<.001	2.319	2.939
Ritual - yes	-.191	.060	-3.176	.002	-.309	-.073
Ritual - no	0	.	.	.	.	.
Bond	.012	.011	1.076	.282	-.010	.034
Loss in past month – yes	-.010	.047	-.211	.833	-.102	.082
Loss in past month - no	0	.	.	.	.	.
Resilience	-.354	.032	-11.030	<.001	-.417	-.291
Age 29 and younger	.336	.111	3.039	.002	.119	.553
Age 30-39	.282	.099	2.849	.004	.088	.476
Age 40-49	.086	.096	.894	.371	-.103	.275
Age 50 and older	0	.	.	.	.	.
Time in field – 11 month/less	-.358	.136	-2.631	.009	-.624	-.091
Time in field – 1-4 years	-.216	.103	-2.092	.037	-.419	-.013
Time in field – 5-9 years	-.187	.102	-1.841	.066	-.386	.012
Time in field – 10-19 years	-.102	.094	-1.087	.277	-.287	.082
Time in field – 20+	0	.	.	.	.	.
LGBTQ+ yes	.028	.059	.476	.634	-.087	.143
LGBTQ+ no	0	.	.	.	.	.

#### 3.8.3 Depression

The multiple linear regression predicting depression was significant (*F*_(12)_ = 16.34, *p* < 0.001), with an *R^2^* of 0.171. Significant predictors of depression included Ritual (B = -0.337; *p* = 0.017; higher Depression reported by those with no ritual), Bond (B = 0.095, *p* <.001; higher depression reported by those with a stronger bond) Resilience (B = -0.872, *p* <.001 higher depression reported by those with lower Resilience), and LGBTQ+ (B = 0.292, *p* = .033; higher depression scores reported by those who identified as LGBTQ+) (**Table 10**).

**Table 10:** Results of the multiple linear regression model predicting Depression as a function of animal loss within past month, bond score, resiliency score, formal process/ritual following death, age, length of time working in the field, and identification as LGBTQ+.

ANOVA						
Model	Sum of Squares	df	Mean Squares	F	Sig.	
Regression Total	556.05 18972.00	12 966	46.34	16.34	<0.001	
Coefficients (Dependent Variable: Depression)					95.0% CI	
Variable	Coefficient (B)	Std. Error	t	Sig.	Lower Bound	Upper Bound
(Constant)	6.241	.370	16.852	<.001	5.514	6.968
Ritual - yes	-.337	.141	-2.389	.017	-.614	-.060
Ritual - no	0	.	.	.	.	.
Bond	.095	.026	3.604	<.001	.043	.147
Loss in past month – yes	.002	.110	.022	.982	-.213	.218
Loss in past month - no	0	.	.	.	.	.
Resilience	-.872	.075	-11.580	<.001	-1.020	-.724
Age 29 and younger	.003	.259	.012	.990	-.505	.512
Age 30-39	-.147	.232	-.633	.527	-.602	.308
Age 40-49	-.115	.226	-.508	.612	-.558	.328
Age 50 and older	0	.	.	.	.	.
Time in field – 11 month/less	.397	.319	1.246	.213	-.228	1.022
Time in field – 1-4 years	.303	.242	1.249	.212	-.173	.778
Time in field – 5-9 years	.235	.238	.986	.324	-.232	.702
Time in field – 10-19 years	.296	.220	1.344	.179	-.136	.729
Time in field – 20+	0	.	.	.	.	.
LGBTQ+ yes	.292	.137	2.129	.033	.023	.562
LGBTQ+ no	0	.	.	.	.	.

#### 3.8.4 Anxiety

The multiple linear regression predicting anxiety was significant (*F*_(12)_ = 18.37, *p* < 0.001), with an *R^2^* of 0.188. Significant predictors of anxiety included Bond (B = 0.067, *p* =.012; higher anxiety reported by those with a stronger bond) and Resilience (B = -0.969, *p* <.001; higher anxiety reported by those with lower Resilience) (**Table 11**).

**Table 11:** Results of the multiple linear regression model predicting Anxiety as a function of animal loss within past month, bond score, resiliency score, formal process/ritual following death, age, length of time working in the field, and identification as LGBTQ+.

ANOVA						
Model	Sum of Squares	df	Mean Squares	F	Sig.	
Regression Total	642.73 23801.00	12 966	53.56	18.37	<0.001	
Coefficients (Dependent Variable: Anxiety)					95.0% CI	
Variable	Coefficient (B)	Std. Error	t	Sig.	Lower Bound	Upper Bound
(Constant)	7.148	.375	19.037	<.001	6.411	7.885
Ritual - yes	-.181	.143	-1.264	.207	-.461	.100
Ritual - no	0	.	.	.	.	.
Bond	.067	.027	2.511	.012	.015	.119
Loss in past month – yes	-.100	.112	-.897	.370	-.319	.119
Loss in past month - no	0	.	.	.	.	.
Resilience	-.969	.076	-12.699	<.001	-1.119	-.820
Age 29 and younger	.335	.263	1.275	.203	-.181	.850
Age 30-39	.211	.235	.897	.370	-.251	.672
Age 40-49	.265	.229	1.156	.248	-.184	.714
Age 50 and older	0	.	.	.	.	.
Time in field – 11 month/less	.323	.323	.999	.318	-.311	.956
Time in field – 1-4 years	.325	.246	1.324	.186	-.157	.808
Time in field – 5-9 years	.148	.241	.615	.539	-.325	.622
Time in field – 10-19 years	-.063	.223	-.284	.777	-.502	.375
Time in field – 20+	0	.	.	.	.	.
LGBTQ+ yes	.171	.139	1.227	.220	-.102	.444
LGBTQ+ no	0	.	.	.	.	.

#### 3.8.5 Perceived organizational support

The multiple linear regression predicting POS was significant (*F*_(12)_ = 13.56, *p* < 0.001, *R^2^* =0.146). Significant predictors of POS included Ritual (B = 2.71, p <.001; higher POS reported by those with a ritual), Bond (B= -0.272, p=.016; lower POS reported by those with a stronger bond) Resilience (B = 2.63, *p* <.001; higher POS reported by those with higher Resilience), Age (B = -4.22, -2.89, *p* <.001; lower POS reported by participants 39 years of age or younger compared to those 40 years of age and older), and time in the field (B = 3.07, *p* =.016; higher POS reported by those in the field either 11 months/less or 20 years or more when compared to those in the field between 1-19 years) (**Table 12**).

**Table 12:** Results of the multiple linear regression model predicting Perceived Organizational Support as a function of animal loss within past month, bond score, resiliency score, formal process/ritual following death, age, length of time working in the field, and identification as LGBTQ+.

ANOVA						
Model	Sum of Squares	df	Mean Squares	F	Sig.	
Regression Total	8434.58 413650.00	12 966	702.88	13.56	<0.001	
Coefficients (Dependent Variable: Perceived Organizational Support)					95.0% CI	
Variable	Coefficient (B)	Std. Error	t	Sig.	Lower Bound	Upper Bound
(Constant)	14.060	1.583	8.882	<.001	10.953	17.166
Ritual - yes	2.712	.603	4.499	<.001	1.529	3.895
Ritual - no	0	.	.	.	.	.
Bond	-.272	.113	-2.420	.016	-.493	-.051
Loss in past month – yes	-.641	.470	-1.362	.174	-1.564	.282
Loss in past month - no	0	.	.	.	.	.
Resilience	2.630	.322	8.173	<.001	1.999	3.262
Age 29 and younger	-4.216	1.107	-3.807	<.001	-6.389	-2.043
Age 30-39	-2.890	.992	-2.914	.004	-4.836	-.944
Age 40-49	-1.025	.965	-1.063	.288	-2.919	.869
Age 50 and older	0	.	.	.	.	.
Time in field – 11 month/less	3.072	1.362	2.256	.024	.399	5.744
Time in field – 1-4 years	1.202	1.036	1.160	.246	-.831	3.235
Time in field – 5-9 years	.272	1.017	.268	.789	-1.723	2.268
Time in field – 10-19 years	-.554	.942	-.588	.556	-2.403	1.295
Time in field – 20+	0	.	.	.	.	.
LGBTQ+ yes	-.761	.587	-1.297	.195	-1.912	.390
LGBTQ+ no	0	.	.	.	.	.

## 4 Discussion

Despite increased recognition of burnout and mental health problems among ACHPs in other work settings (animal shelters, veterinary practice), the wellbeing of those who work and/or volunteer in zoo settings has received minimal research attention. This study addresses this gap, giving us a better understanding of U.S. zoo staff and volunteers’ job fulfillment, POS, burnout, mental health, resiliency, and experiences of animal loss. Our findings suggest that ACHPs in zoo settings face similar struggles as those in other settings, indicating a need to prioritize supportive services for ACHPs within zoo settings.

### 4.1 Job fulfillment and perceived organizational support

Only 31% of the total sample exceeded the cut-off for professional fulfillment. ACHPs reported the lowest levels of professional fulfillment (25%), significantly lower than other staff (42%), or volunteers (68%). Our findings mirror prior studies among ACHPs in other settings that have reported low rates of professional fulfillment (11, 49). Given the association between professional fulfillment and levels of well-being and burnout among ACHPs (11, 49), investing in programs, policies, and practices that better support staff, particularly ACHPs, in their professional development is key for zoos that want to prioritize employee wellbeing.

We also found evidence of differences in POS by role. Volunteers reported feeling the most organizational support, followed by other staff, and then AHCPs. While possible POS scores ranged from 7 to 35; the average score for ACHPs was 19 (compared to 23 for Others and 28 for Volunteers). These scores suggest that zoos have an opportunity to improve their supportive services. Because prior research suggests POS is negatively associated with burnout, grief, stress, and several indicators of poor mental health (8, 42, 46–48), investing resources in strengthening and maintaining perceptions of organizational support may have benefits to both zoo professionals and volunteers.

### 4.2 Burnout, mental health, and resiliency

In the current study, we found that ACHPs, compared to other staff and volunteers, reported the highest rates of burnout. A majority (55%) of ACHPs scored at or above the burnout threshold, compared to 32% of other staff and 5% of volunteers. This finding is consistent with prior research indicating higher burnout scores in careers that include higher degrees of animal contact (69). The rate of burnout among ACHPs in the current study is comparable to those reported for animal shelter and veterinary professionals (e.g., (11, 54, 70). This rate also mirrors the estimated percentage of human medical professionals who experience burnout, with most estimates exceeding 50% (11, 71–73).

Our findings support prior evidence that ACHPs are at increased risk for anxiety and depression. In the general U.S. population, the estimated rate of past-year anxiety disorder diagnoses (of any type) and past-year major depressive episode is estimated to be 19.1% and 8.3%, respectively (74–76). In the current sample, 43% of ACHPs met or exceeded our “yellow flag” cutoff for the presence of anxiety, providing initial evidence that rates of anxiety may be higher among zoo ACHPs than in the general population. We also found that the rate of moderate to severe anxiety was 32% and 5% for other staff and volunteers, respectively. Thus, other zoo staff, regardless of having a direct role in animal care/health, demonstrate elevated rates of anxiety. Similar patterns were found for depression. 33% of ACHPs and 22% of other staff met our cut-point for the presence of depression, whereas only 4% of volunteers met this criterion.

When we assessed resilience, we found that 24% of participants had scores indicating low resilience, 66% were in the normative range, and 10% had scores indicating high resilience. More specifically, 10% of ACHPs’ scores suggested high resilience, compared to 8% of Others and 17% of Volunteers. Accordingly, a notable proportion of paid staff could benefit from opportunities to develop or enhance their adaptive coping strategies to foster resilience in the workplace. Results of our regression analyses provide further support for this assertion. Our findings indicated that higher levels of resiliency were associated with lower levels of burnout, depression, and anxiety, and higher levels of professional fulfillment and POS. Given that only 10% of ACHPs and 8% of other staff scored in the high resilience category, our findings suggest that investing in helping zoo professionals develop resiliency through adaptive coping is an important area of opportunity for zoos who aim to promote employee wellbeing. In addition, there is increasing recognition that resiliency is also something that can be fostered on a team level. Team resilience has been defined as the processes of “managing pressure effectively across the team as a whole [ … ], that further strengthen the capacity of the team to deal with future challenges in adversity” (77). The premise of team resilience is that adverse stressors can negatively affect team members’ health and team performance, and as a result, impact a team’s overall functioning level (78). While most stress research has focused on individuals’ stress and resiliency, many organizations are recognizing the need to support teams, especially during and after adverse events (79). Zoos that prioritize team and individual resilience could impact not only their employees’ mental health, but the functionality of the organization.

### 4.3 Animal bonds, animal loss, and rituals

In addition to job fulfillment, POS, and mental health, we also examined participants’ bonds with the animals they care for. We found that reported levels of bond were relatively consistent regardless of the type of species/taxa cared for by participants. For ACHPs, the mean level of reported bond (range 1-10) across all four types of losses (anticipated, unexpected, transfer, job change) ranged from 6.06 to 8.15. The mean bond for other staff ranged from 4.86 to 7.25 and volunteer bond means ranged from 4.64 to 6.0. Thus, regarding experiences of animal loss, individuals who worked closely with animals reported, on average, higher bonds with these animals. Furthermore, results of our regression analyses showed that level of ACHP’s bond with animals was positively and significantly associated with depression and anxiety, such that those with stronger bonds had higher levels of depression and anxiety.

Results of this study identified the most common losses experienced by ACHPs in the past year were unexpected death (experienced by 80%) and anticipated loss (experienced by 74%), with more than half of these losses occurring within the past 3 months. These types of death were also the most frequently reported types of loss for other staff and volunteers (37% and 50%, respectively). A larger percentage of other staff and volunteers, compared to ACHPs, reported not having experienced these types of losses (within the past year or ever at their current zoological institution). Among those who had not experienced a loss in the past year, 80% reported having experienced an unexpected death or anticipated death at some point.

Although animal loss is a common experience for ACHPs, only 17% of participants in our sample (16.6% of ACHPs) indicated that their zoo had a formal process or ritual that was performed following the death of an animal. This finding is important given results of the regression models suggesting that although animal loss is not a significant predictor of any outcome examined in the current study, having a formal ritual or process is positively associated with job fulfillment and POS and negatively associated with depression and burnout. This suggests that despite the high prevalence of loss experiences among ACHPs, how these losses are handled may be more important in the context of ACHPs’ wellbeing. Additionally, our findings concerning participants’ bonds with animals highlight that *all* animal loss is important, regardless of species. Given that higher value and attention tends to be placed on charismatic animals, such as small and large mammals (80, 81), it is important that zoos practice equity in honoring experiences of loss among those who work with all species, including fish, amphibians, and reptiles.

### 4.4 Demographic considerations

In the current study, we found that 20% of study participants identified as LGBTQ+. Prior research suggests that LGBTQ+ people make up approximately 6% of the U.S. workforce (81); moreover, representation of LGBTQ+ identities in the general U.S. population is estimated at 7% (82, 83). Thus, the representation of LGBTQ+ people in the current study is an important finding. There are few potential explanations for the higher-than-expected rate of LGBTQ+ representation in the current study. For example, prior studies indicate a high degree of orientation towards animals, and value placed on animals, among members of the LGBTQ+ community (84–88). There is also some evidence that LGBTQ+ individuals, particularly early career workers, report lower salary expectations than heterosexual individuals and are more likely to embrace “altruistic” work values and to indicate a career choice in the nonprofit sector (89). Thus, working in zoos may be particularly appealing to some members of the LGBTQ+ community.

Results of this study also suggest that LGBTQ+ status is positively associated with depression among ACHPs, even when adjusting for the effects of participant age, time in the field, experiences of animal loss, bonds with animals, and individual resiliency. This finding is not surprising given that exposure to minority stressors results in increased levels of depression in the LGBTQ+ population, who, overall, are impacted by disproportionate rates of mental health issues when compared to their cisheterosexual peers (84, 90). Prior research also suggests that LGBTQ+ workers with poor or neutral mental health have greater odds of working in low-wage sectors (91). The issue of low pay and living wages for those working in zoo settings has been an ongoing topic of discussion in the industry (39, 92–96); however, more contemporary, rigorous and representative data are needed to support these claims. To promote equity within the industry, future research on the experiences and wellbeing of LGBTQ+ people in zoo settings is warranted. We recommend that future studies in this area capture socioeconomic data in tandem with LGBTQ+ workers’ mental health and explore the representation of LGBTQ+ identities across all levels of zoos’ organizational structure (e.g., formal leadership roles, managers vs. non-managers, hourly staff) to test whether LGBTQ+ representation is equitable across job function and pay grades. Given the limitations of the current sample concerning other forms of demographic diversity, research intentionally designed to identify the representation and experiences of individuals with other marginalized identities in the zoo industry (e.g., racialized staff and/or those with disabilities) is also an important direction for future research (97).

Our results also suggest that ACHPs’ age and time in the field are important demographic factors that warrant further attention in relation to zoo professionals’ wellbeing. Results of our regression model predicting burnout indicated that ACHPs ages 39 and younger may be most at risk for burnout, compared to staff who are 40 years of age or older. However, it is possible that this result is confounded by individuals’ position or role within the organization. Individuals in the 40+ age group may be more likely to have leadership positions that afford more agency and control in the workplace, thereby contributing to lower levels of burnout in the current sample (7, 21, 31). We recommend that future studies assess these factors in more detail. Age was also a significant predictor in our regression model predicting POS, which indicated that participants 39 years of age or younger, compared to those 40 years of age and older, reported lower levels of organizational support. Regarding age group differences, it is interesting to consider that individuals in the current sample who are 18 to 29 years of age represent Gen Z and Millennials, whereas the 40+ groups reflect individuals on the Millennial/Gen X cusp, Gen X, and Baby Boomers. Thus, it is possible that generational differences may influence perceptions of organization support (8, 98) or that ways that zoos invest in and demonstrate support for staff are more aligned with older generations. Lastly, ACHPs who have been in the field either 6 to 11 months or 20 years or more had higher levels of perceived organization support compared to those in the field 1-19 years. One potential explanation for this finding is that staff who are in their first year on the job are still in the onboarding process and perceive a higher degree of support due to organizational efforts to integrate them into their new role. Moreover, individuals who have been in the field more than 20 years may be those whose values and occupational needs are well aligned with the culture of their organization. Our results suggest that better understanding the organizational support needs of early and mid-career ACHP is an important direction for future inquiry.

### 4.5 Implications and future directions for research

Promoting mental health among all employees and volunteers, but especially ACHPs, in zoo settings is not only important for enhancing their wellbeing but could also help to mitigate the potentially harmful impact of poor mental health and burnout on the health and welfare of animals under human care. Although the association between physician burnout, professional inefficiencies, and suboptimal patient care is well documented in human medicine (99), less is known about the impact of ACHPs’ burnout on the animals under their care. Brando et al. (39) surveyed zoo and aquarium professionals and identified common themes regarding these professionals’ lack of ability to feel empowered to do their best for animal welfare. The study also identified links between staff welfare and perceptions of animal welfare and suggested that by taking better care of their people via reduction of stressors, zoos can improve the ability of their staff to care for animals. Future research should explore whether programs that aim to support ACHP wellbeing (e.g., GRAZE [Growing Resiliency in Aquarium and Zoo Employees]) have indirect effects on the wellbeing of animals under human care in zoos. Furthermore, it will be important to assess if this effect is evident when utilizing objective measures of animal health and wellbeing (e.g., biomarkers), as well as zoo guests’ perceptions of animal care. Additionally, research suggests associations between professional burnout and general safety compliance (100). Because zoo-based ACHPs work with captive wildlife that may pose risks to staff and guest safety, as well as machinery and heavy equipment, understanding the links between burnout, mental health, and occupational safety and compliance in the zoo industry are also important directions for future research.

By offering Employee Assistance Programs (EAPs), zoos can provide confidential access to professional counseling services for staff. These programs can offer problem assessment, short-term counseling, and referrals to appropriate community and private services. In situations where ongoing and long-term counseling is needed, ensuring costs are covered by health insurance plans with co-pay fees that are affordable for zoo staff and do not cause unnecessary financial burden is key and has important implications for fostering equitable access to mental health support services. Unfortunately, EAPs are often underutilized by employees (101, 102). A lack of knowledge about mental health and concomitant stigma toward mental health problems and help-seeking behavior often results in delays in seeking professional support via EAPs. Others may associate EAP programs as a resource for coping with personal rather than professional challenges (101). Sometimes the emotional energy resulting from moral distress or workplace trauma prohibits people from taking initial steps in seeking support (103). Increasing awareness about the opportunities available through EAPs and streamlining processes could decrease barriers to access. Brokering connections for zoo employees to counselors with expertise in zoo-related loss and grief may foster validation of grief responses and enhance POS. Future research is needed to establish rates of mental health stigma and attitudes toward help-seeking behavior among zoo professionals.

In this vein, it is also important for the zoo industry to recognize that zoo professionals’ access to appropriate and timely services is further compounded by the scarcity of behavioral and mental health professionals. A 2022 survey of mental health practitioners conducted by the American Psychological Association found that 60% of psychologists reported no openings for new patients and more than 40% had a waiting list of 10 patients or more (104). Notably, the U.S. Department of Health and Human Services estimates a deficit of 10,000 mental health professionals by 2025 across the country, highlighting the urgent need for innovative approaches to promote mental health. Workplaces are increasingly recognized as effective places to promote mental health literacy programs and other health promotion activities (105). Mental health literacy refers to an individual’s knowledge and beliefs about mental disorders, which aids in the recognition, management or prevention of mental health problems and reduces stigma about mental health and help-seeking. Not only is mental health literacy recognized as a method and tool for creating a mentally healthy and resilient organizational culture, but it is also increasingly recognized as an aspect of leader competency (106).

Given the increased risk for common mental health problems among ACHPs, improving mental health literacy in zoo leadership and people managers may support early identification of distress and related psychological health concerns among zoo staff and aid in facilitating help-seeking behavior. Specifically, standardized, psychoeducational programs that combat mental health problems and suicide may be effective models to employ in zoo settings. Curriculum programs such as Mental Health First Aid (MHFA), which teach program participants how to combat stigmatizing attitudes toward mental health, recognize acute mental health crises in others, and connect peers with helpful resources, may be particularly effective. MHFA has been adopted in more than 20 other countries around the world and has been evaluated in several studies, a majority of which have shown the MHFA program is effective in improving mental health knowledge, reducing stigmatizing attitudes, and increasing supportive behaviors (107).

Findings from this study elevate the need for both individual zoos and other organizations (e.g., AZA, Zoological Association of America, America Association of Zookeepers, World Association of Zoos and Aquariums) to advance practices regarding loss and grief surrounding animal deaths and transfers. Ideally, zoos would move towards cultures which promote open discussion of loss and grief and the integration of rituals prior to loss and into bereavement; thus, reducing disenfranchised grief responses. Despite differences in human and animal care settings, professionals who experience significant human loss can offer direction for creating proactive grief response cultures. Based on a study of occupational therapists’ workplace bereavement experiences, Gilbert et al. (108) developed the C.A.R.E. Model of Employee Bereavement Support as a framework for organizations to respond to workplace loss and grief. The model incorporates four key components: (1) an emphasis on open, two-way *communication* about the loss; (2) the provision of *accommodations* to support employee’s individual needs such as adapting work demands or hours; (3) *recognition* and acknowledgment of the impact of the loss at the individual and organizational level; and (4) offering *emotional support* such as empathic responses from managers, peers, or consumers.

As evidenced from this study’s national sample of zoo employees and volunteers, rituals are negatively associated with burnout, depression, and anxiety. Fortunately, building grief rituals can be done with limited financial and human resources, providing opportunities for employees to invest in activities that feel meaningful in their healing. For example, in preparation for an anticipated loss due to transfer or death, allowing the space and time for employees to say “goodbye” to animals prior to the loss and participating in activities that foster a positive death experience may reduce negative grief reactions such as guilt about not being present for the animal at the end of life. However, circumstances surrounding death, including sudden deaths, staffing shortages, or individual comfort in leading grief rituals, may limit opportunities for pre-loss activities. In these situations, as with any animal death, opening spaces for celebration of life rituals, whether at regular team meetings or designated grief ceremonies, would promote the beneficial practice of continuing bond expressions such as sharing memories, photographs, or other meaningful objects (109–111). Preferably, organizations should allow for flexibility in their grief programming to support individual needs and be responsive to losses which may be experienced as traumatic, complicated, or occur in succession. Lastly, enhancing organizational grief resiliency skills through psychoeducation about loss and grief, including educational readings, webinars, or invited presentations by contracted grief counselors, could enhance individual and collective resilience and reduce the risk of complicated grief trajectories (112).

### 4.6 Limitations

Limitations of this study include that our survey was only available in the English language, and that we relied on a cross-sectional convenience sampling strategy. Furthermore, a majority of our sample identified as cisgender, white women. Although this finding is relatively consistent with publicly available demographic data on the zoo industry (113), because of this limited diversity, we did not have adequate statistical power to examine potential differences between or within racial and ethnic groups. Given evidence of disparities in mental health and access to mental health services between white and minoritized racial/ethnic groups (due to the impacts of systemic and structural racism on racialized population groups), as well as disproportionate rates of workplace discrimination and microaggressions experienced by marginalized individuals/communities, future research should explore variations in rates of burnout, mental health, POS and professional fulfillment across racial and ethnic identities within the zoo industry. It is important to understand whether there are racially-, ethnically-, and/or other identity-specific (sexual orientation, gender identity, disability, socioeconomic status) factors associated with these outcomes among zoo staff and volunteers. Such research could help to inform inclusive, culturally responsive interventions and promote equity in the workplace.

Other limitations of this study include that we did not assess cumulative animal losses within a specific period of time. Given the psychological impacts of complex (compounded) trauma, this is an important direction for future research. In addition, a limitation of our study is the potential lack of consistent methods used to recruit participants at other zoos. For example, it is possible that only ACHPs may have received the survey at some zoos, while participants of varying roles were recruited at others. In other words, although our study was open to any zoo staff member or volunteer who had worked at their current zoo for 6 months, zoo leaders who assisted us with recruitment may have only sent the recruitment flyer to individuals or teams for whom animal loss was most relevant. This may have contributed to the large number of ACHPs and prevalence of recent animal loss in the current study. Relatedly, our utilization of AZA forums for recruitment may have contributed to bias in our sample; we are unable to determine how many individuals chose not to participate in our study and if there are any systematic differences in rates of participation across groups (e.g., avian vs. ungulate group forums). In addition, for participants who reported animal loss(es), we did not account for the length of their relationship with the animal or the animal’s species or taxa in our analyses. Future research should test whether duration of the relationship and/or type of animal moderates the association between animal loss and the mental health outcomes explored in this study. It is possible that ACHPs who work with popular animals (e.g., mammals) have different experiences of animal loss than those who care for lesser-known or less “popular” animals due to varying levels of value placed on different species and taxa.

### 4.7 Conclusion

Our study provides compelling data indicating that the wellbeing of ACHPs in zoo settings is a significant concern with potential negative impacts on individuals, organizations, and animals under their care. Specifically, our findings suggest that ACHPs in zoos are struggling with disproportionate rates of burnout, anxiety, depression, and low rates of professional fulfilment and perceived organizational support. Zoos should develop organizational plans that foster a culture which normalizes and validates grief/loss experiences and is proactive in responding to animal loss, related trauma, and other occupational stressors. Building organizational resilience and offering opportunities for staff to develop adaptive coping and individual resiliency will be important actions in this effort. The results of this research sound the call for systemic changes within the zoo industry, for the betterment and welfare of both humans and the animals under their care.

## Data availability statement

The datasets for this article are not publicly available due to concerns regarding participant anonymity. Requests to access the datasets should be directed to smcdonald@denverzoo.org.

## Ethics statement

This study was approved by Colorado State University Institutional Review Board (IRB #4770). This study was conducted in accordance with the local legislation and institutional requirements. Written informed consent for participation was not required from the participants because the study was categorized as exempt by Colorado State University’s Institutional Review Board. Because this was an anonymous survey, written informed consent was not required. An introductory statement explained the study and indicated to potential participants that consent was implied by completing the survey.

## Author contributions

SM: Conceptualization, Investigation, Methodology, Project administration, Resources, Writing – original draft, Writing – review & editing. LK: Conceptualization, Data curation, Formal analysis, Investigation, Methodology, Writing – original draft, Writing – review & editing. NN: Conceptualization, Data curation, Writing – review & editing. JC-M: Conceptualization, Writing – original draft, Writing – review & editing. RD-M: Data curation, Writing – review & editing.

## References

[1] Fink-Miller EL Nestler LM. Suicide in physicians and veterinarians: risk factors and theories. Curr Opin Psychol. (2018) 22:23–6. doi: 10.1016/j.copsyc.2017.07.019 30122273

[2] Kollias NS Strand EB Kogan LR Houlihan KE Thompson-Iritani S Hoenig DE. Psychological implications of humane endings on the veterinary profession. J Am Veterinary Med Assoc. (2023) 261:185–92. doi: 10.2460/javma.22.06.0234 36701216

[3] Marton B Kilbane T Nelson-Becker H. Exploring the loss and disenfranchised grief of animal care workers. Death Stud. (2020) 44:31–41. doi: 10.1080/07481187.2018.1519610 30654733

[4] Merk Animal Health. Significant Study on Veterinary Wellbeing Reveals importance of continued focus on personal and professional health and well-being among veterinarians (2020). Available online at: https://www.merck-animal-health-usa.com/newsroom/wellbeing-study-2020 (Accessed October 13, 2023).

[5] Moir FM Van den Brink ARK. Current insights in veterinarians’ psychological wellbeing. New Z Veterinary J. (2020) 68:3–12. doi: 10.1080/00480169.2019.1669504 31607215

[6] Perret JL Best CO Coe JB Greer AL Khosa DK Jones-Bitton A. Association of demographic, career, and lifestyle factors with resilience and association of resilience with mental health outcomes in veterinarians in Canada. J Am Vet Med Assoc. (2020) 257:1057–68. doi: 10.2460/javma.2020.257.10.1057 33135980

[7] Volk JO Schimmack U Strand EB Lord LK Siren CW. Executive summary of the merck animal health veterinary wellbeing study. J Am Vet Med Assoc. (2018) 252:1231–8. doi: 10.2460/javma.252.10.1231 29701527

[8] Paul NK Cosh SM Lykins AD. “A love–hate relationship with what I do”: protecting the mental health of animal care workers. Anthrozoös. (2023) 36:489–508. doi: 10.1080/08927936.2023.2166712

[9] American Veterinary Medical Association. Wellbeing resources for veterinary professionals . Available online at: https://www.avma.org/resources-tools/wellbeing (Accessed January 10, 2024).

[10] Hill EM LaLonde CM Reese LA. Compassion fatigue in animal care workers. Traumatology. (2020) 26:96–108. doi: 10.1037/trm0000218

[11] Kogan LR Wallace JE Schoenfeld-Tacher R Hellyer PW Richards M. Veterinary technicians and occupational burnout. Front Vet Sci. (2020) 7:328. doi: 10.3389/fvets.2020.00328 32596271 PMC7303959

[12] Scotney RL McLaughlin D Keates HL. An investigation of the prevalence of compassion fatigue, compassion satisfaction and burnout in those working in animal-related occupations using the Professional Quality of Life (ProQoL) Scale. Vet Nurse. (2019) 10:276–84. doi: 10.12968/vetn.2019.10.5.276

[13] Chang FT Hart LA. Human-animal bonds in the laboratory: How animal behavior affects the perspective of caregivers. ILAR J. (2002) 43:10–8. doi: 10.1093/ilar.43.1.10 11752726

[14] LaFollette MR Riley MC Cloutier S Brady CM O’Haire ME Gaskill BN. Laboratory animal welfare meets human welfare: A cross-sectional study of professional quality of life, including compassion fatigue in laboratory animal personnel. Front Vet Sci. (2020) 7:114. doi: 10.3389/fvets.2020.00114 32195275 PMC7066073

[15] Newsome JT Clemmons EA Fitzhugh DC Gluckman TL Creamer-Hente MA Tambrallo LJ. Compassion fatigue, euthanasia stress, and their management in laboratory animal research. J Am Assoc Lab Anim Sci. (2019) 58:289–92. doi: 10.30802/AALAS-JAALAS-18-000092 PMC6526492 31014414

[16] Andrukonis A Protopopova A. Occupational health of animal shelter employees by live release rate, shelter type, and euthanasia-related decision. Anthrozoös. (2020) 33:119–31. doi: 10.1080/08927936.2020.1694316

[17] Hoy-Gerlach J Ojha M Arkow P. Social workers in animal shelters: A strategy toward reducing occupational stress among animal shelter workers. Front Vet Sci. (2021) 8:734396. doi: 10.3389/fvets.2021.734396 34859083 PMC8631323

[18] Jacobs J Reese LA. Compassion fatigue among animal shelter volunteers: examining personal and organizational risk factors. Anthrozoös. (2021) 34:803–21. doi: 10.1080/08927936.2021.1926719

[19] Black AF Winefield HR Chur-Hansen A. Occupational stress in veterinary nurses: Roles of the work environment and own companion animal. Anthrozoös. (2011) 24:191–202. doi: 10.2752/175303711X12998632257503

[20] Fournier AK Mustful B. Compassion fatigue: Presenting issues and practical applications for animal-caring professionals. In: Kogan L Blazina C, editors. Clinician’s Guide to Treating Companion Animal Issues. Cambridge, MA, USA: Academic Press (2019). p. 511–34.

[21] Hatch PH Winefield HR Christie BA Lievaart JJ. Workplace stress, mental health, and burnout of veterinarians in Australia. Aust Veterinary J. (2011) 89:460–8. doi: 10.1111/avj.2011.89.issue-11 22008127

[22] Moore IC Coe JB Adams CL Conlon PD Sargeant JM. The role of veterinary team effectiveness in job satisfaction and burnout in companion animal veterinary clinics. J Am Veterinary Med Assoc. (2014) 245:513–24. doi: 10.2460/javma.245.5.513 25148093

[23] Nett RJ Witte TK Holzbauer SM Elchos BL Campagnolo ER Musgrave KJ. Risk factors for suicide, attitudes toward mental illness, and practice-related stressors among US veterinarians. J Am Veterinary Med Assoc. (2015) 247:945–55. doi: 10.2460/javma.247.8.945 26421408

[24] Reeve CL Rogelberg SG Spitzmüller C DiGiacomo N. The caring-killing paradox: euthanasia-related strain among animal-shelter workers 1. J Appl Soc Psychol. (2005) 35:119–43. doi: 10.1111/j.1559-1816.2005.tb02096.x

[25] Rohlf VI. Interventions for occupational stress and compassion fatigue in animal care professionals—A systematic review. Traumatology. (2018) 24:186. doi: 10.1037/trm0000144

[26] Stetina BU Krouzecky C. Reviewing a decade of change for veterinarians: past, present and gaps in researching stress, coping and mental health risks. Animals. (2022) 12:3199. doi: 10.3390/ani12223199 36428425 PMC9686667

[27] Scotney RL McLaughlin D Keates HL. A systematic review of the effects of euthanasia and occupational stress in personnel working with animals in animal shelters, veterinary clinics, and biomedical research facilities. J Am Veterinary Med Assoc. (2015) 247:1121–30. doi: 10.2460/javma.247.10.1121 26517615

[28] White DJ Shawhan R. Emotional responses of animal shelter workers to euthanasia. J Am Veterinary Med Assoc. (1996) 208:846–9. doi: 10.2460/javma.1996.208.06.846 8617639

[29] Bartram DJ Baldwin DS. Veterinary surgeons and suicide: influences, opportunities and research directions. Veterinary Rec. (2008) 162:36–40. doi: 10.1136/vr.162.2.36 18192654

[30] Chan CK Wong PW. Predictors of suicide risk and mental health outcomes among Hong Kong veterinarians: a cross-sectional study. Behav Sci. (2023) 13:770. doi: 10.3390/bs13090770 37754048 PMC10526002

[31] Platt B Hawton K Simkin S Mellanby RJ. Suicidal behaviour and psychosocial problems in veterinary surgeons: a systematic review. Soc Psychiatry Psychiatr Epidemiol. (2012) 47:223–40. doi: 10.1007/s00127-010-0328-6 21181111

[32] Tomasi SE Fechter-Leggett ED Edwards NT Reddish AD Nett RJ. All causes of death among veterinarians in the United States during 1979 through 2015. J Am Veterinary Med Assoc. (2022) 260:1–0. doi: 10.2460/javma.21.04.0221 36927951

[33] Tomasi SE Fechter-Leggett ED Edwards NT Reddish AD Crosby AE Nett RJ. Suicide among veterinarians in the United States from 1979 through 2015. J Am Veterinary Med Assoc. (2019) 254:104–12. doi: 10.2460/javma.254.1.104 PMC6417412 30668293

[34] Schwerdtfeger KA Bahramsoltani M Spangenberg L Hallensleben N Glaesmer H. Depression, suicidal ideation and suicide risk in German veterinarians compared with the general German population. Veterinary Rec. (2020) 186:e2. doi: 10.1136/vr.105430 32229508

[35] Witte TK Spitzer EG Edwards N Fowler KA Nett RJ. Suicides and deaths of undetermined intent among veterinary professionals from 2003 through 2014. J Am Veterinary Med Assoc. (2019) 255:595–608. doi: 10.2460/javma.255.5.595 PMC6933287 31429646

[36] Platt B Hawton K Simkin S Mellanby RJ. Systematic review of the prevalence of suicide in veterinary surgeons. Occup Med. (2010) 60:436–46. doi: 10.1093/occmed/kqq044 20591857

[37] Kramper S Crosby ES Waitz-Kudla SN Weathers F Witte TK. Highly stressful events and posttraumatic stress disorder symptoms among veterinary professionals: Prevalence and associations with mental health and job-related outcomes. psychol Trauma: Theory Research Practice Policy. (2023) 15:S275–85. doi: 10.1037/tra0001432 36689377

[38] Bemister-Bourret K Tawfik M. Record, recall, reflect: a qualitative examination of compassion fatigue in Toronto Zoo staff. J Zoological Botanical Gardens. (2023) 4:413–26. doi: 10.3390/jzbg4020031

[39] Brando S RaChinas-Lopes P Goulart VD Hart LA. Understanding job satisfaction and occupational stressors of distinctive roles in zoos and aquariums. Animals. (2023) 13:2018. doi: 10.3390/ani13122018 37370528 PMC10295341

[40] Marino J. Measuring burnout in zoo & aquarium professionals: a comparative analysis of demographics (2023). Authorea. Available online at: https://d197for5662m48.cloudfront.net/documents/publicationstatus/136748/preprint_pdf/268944da4d93e57ebcdaf30f82aee97d.pdf (Accessed January 11, 2024).

[41] Jackson D Firtko A Edenborough M. Personal resilience as a strategy for surviving and thriving in the face of workplace adversity: a literature review. J Advanced Nurs. (2007) 60:1–9. doi: 10.1111/j.1365-2648.2007.04412.x 17824934

[42] Cushing N Meehan C Norris K. Resilience in animal care professions: does the stress shield model fit? Aust Veterinary J. (2022) 100:513–25. doi: 10.1111/avj.13193 35698265

[43] Mealer M Jones J Newman J McFann KK Rothbaum B Moss M. The presence of resilience is associated with a healthier psychological profile in ICU nurses: Results of a national survey. Int J Nurs Stud. (2012) 49:292–9. doi: 10.1016/j.ijnurstu.2011.09.015 PMC3276701 21974793

[44] Lloyd C Campion DP. Occupational stress and the importance of self-care and resilience: focus on veterinary nursing. Irish Veterinary J. (2017) 70:1–7. doi: 10.1186/s13620-017-0108-7 PMC5613522 29021894

[45] Paton D Violanti JM Johnston P Burke KJ Clarke J Kennan D. Stress shield: A model of police resilience. Int J Emerg Ment Health. (2008) 10:95–108. 18788345

[46] Kogan LR Rishniw M. Differences in perceptions and satisfaction exist among veterinarians employed at corporate versus privately owned veterinary clinics. J Am Veterinary Med Assoc. (2023) 261:1838–46. doi: 10.2460/javma.23.06.0326 37607680

[47] Maslach C Schaufeli WB Leiter MP. Job burnout. Annu Rev Psychol. (2001) 52:397–422. doi: 10.1146/annurev.psych.52.1.397 11148311

[48] West CP Dyrbye LN Shanafelt TD. Physician burnout: Contributors, consequences and solutions. J Internal Med. (2018) 283:516–29. doi: 10.1111/joim.12752 29505159

[49] Powell L Reinhard CL Serpell J Watson B. Workplace relations and opportunities for career development impact the retention of veterinarians in shelter medicine. Front Veterinary Sci. (2021) 8:732105. doi: 10.3389/fvets.2021.732105 PMC8424191 34513978

[50] Wallace JE. Meaningful work and well-being: a study of the positive side of veterinary work. Vet Rec. (2019) 185:571. doi: 10.1136/vr.105146 31563892

[51] Fraser J Clayton S Sickler J Taylor A. Belonging at the zoo: retired volunteers, conservation activism and collective identity. Ageing Soc. (2009) 29:351–68. doi: 10.1017/S0144686X08007915

[52] Kramer MW Danielson MA. Communication and role development for zoo volunteers: responding to role-sending, role-making, and role-remaking. J Appl Communication Res. (2017) 45:96–115. doi: 10.1080/00909882.2016.1248466

[53] Corso AL. The role of work in animal shelter volunteers’ experiences of compassion fatigue. Walden University, Minneapolis (MN (2022).

[54] Monaghan H Rohlf V Scotney R Bennett P. Compassion fatigue in people who care for animals: An investigation of risk and protective factors. Traumatology. (2020). doi: 10.1037/trm0000246

[55] Polachek AJ Wallace JE. The paradox of compassionate work: a mixed-methods study of satisfying and fatiguing experiences of animal health care providers. Anxiety Stress Coping. (2018) 31:228–43. doi: 10.1080/10615806.2017.1392224 29064289

[56] Trockel M Bohman B Lesure E Hamidi MS Welle D Roberts L. A brief instrument to assess both burnout and professional fulfillment in physicians: reliability and validity, including correlation with self-reported medical errors, in a sample of resident and practicing physicians. Acad Psychiatry. (2018) 42:11–24. doi: 10.1007/s40596-017-0849-3 29196982 PMC5794850

[57] Eisenberger R Huntington R Hutchison S Sowa D. Perceived organizational support. J Appl Psychol. (1986) 71:500. doi: 10.1037//0021-9010.71.3.500

[58] Worley JA Fuqua DR Hellman CM. The survey of perceived organisational support: which measure should we use? SA J Ind Psychol. (2009) 35:112–6. doi: 10.4102/sajip.v35i1.754

[59] Cropanzano R Howes JC Grandey AA Toth P. The relationship of organizational politics and support to work behaviors, attitudes, and stress. J Organizational Behavior: Int J Industrial Occup Organizational Psychol Behav. (1997) 18:159–80. doi: 10.1002/(ISSN)1099-1379

[60] Kroenke K Spitzer RL Williams JB Löwe B. An ultra-brief screening scale for anxiety and depression: the PHQ–4. Psychosomatics. (2009) 50:613–21. doi: 10.1016/S0033-3182(09)70864-3 19996233

[61] Löwe B Wahl I Rose M Spitzer C Glaesmer H Wingenfeld K. A 4-item measure of depression and anxiety: validation and standardization of the Patient Health Questionnaire-4 (PHQ-4) in the general population. J Affect Disord. (2010) 122:86–95. doi: 10.1016/j.jad.2009.06.019 19616305

[62] Kazlauskas E Gelezelyte O Kvedaraite M Ajdukovic D Johannesson KB Böttche M. Psychometric properties of the Patient Health Questionnaire-4 (PHQ-4) in 9230 adults across seven European countries: Findings from the ESTSS ADJUST study. J Affect Disord. (2023) 335:18–23. doi: 10.1016/j.jad.2023.05.007 37164064

[63] Lenz AS Li C. Evidence for measurement invariance and psychometric reliability for scores on the PHQ-4 from a rural and predominately hispanic community. Measurement Eval Couns Dev. (2022) 55:17–29. doi: 10.1080/07481756.2021.1906157

[64] Materu J Kuringe E Nyato D Galishi A Mwanamsangu A Katebalila M. The psychometric properties of PHQ-4 anxiety and depression screening scale among out of school adolescent girls and young women in Tanzania: a cross-sectional study. BMC Psychiatry. (2020) 20:1–8. doi: 10.1186/s12888-020-02735-5 32560705 PMC7304148

[65] Tibubos AN Kröger H. A cross-cultural comparison of the ultrabrief mental health screeners PHQ-4 and SF-12 in Germany. psychol Assess. (2020) 32:690. doi: 10.1037/pas0000814 32191078

[66] Caro-Fuentes S Sanabria-Mazo JP. A systematic review of the psychometric properties of the Patient Health Questionnaire-4 (PHQ-4) in clinical and non-clinical populations. J Acad Consultation-Liaison Psychiatry. (2023). doi: 10.1016/j.jaclp.2023.11.685 38012988

[67] Smith BW Dalen J Wiggins K Tooley E Christopher P Bernard J. The brief resilience scale: assessing the ability to bounce back. Int J Behav Med. (2008) 15:194–200. doi: 10.1080/10705500802222972 18696313

[68] Smith BW deCruz-Dixon N Schodt K Torres F. Brief Resilience Scale (BRS). In: Handbook of Assessment in Mindfulness Research. Springer International Publishing, Cham (2023). p. 1–19.

[69] Andrukonis A Hall NJ Protopopova A. The impact of caring and killing on physiological and psychometric measures of stress in animal shelter employees: A pilot study. Int J Environ Res Public Health. (2020) 17:9196. doi: 10.3390/ijerph17249196 33317016 PMC7764342

[70] Ouedraogo FB Lefebvre SL Hansen CR Brorsen BW. Compassion satisfaction, burnout, and secondary traumatic stress among full-time veterinarians in the United States (2016–2018). J Am Vet Med Assoc. (2021) 258:1259–70. doi: 10.2460/javma.258.11.1259 33978434

[71] Lemaire JB Wallace JE. Burnout among doctors: a system level problem requiring a system level response. BMJ. (2017) 358:j3360. doi: 10.1136/bmj.j3360 28710272

[72] Oxtoby C Ferguson E White K Mossop L. We need to talk about error: causes and types of error in veterinary practice. Vet Rec. (2015) 177:438–44. doi: 10.1136/vr.103331 26489997

[73] Reith TP. Burnout in United States healthcare professionals: a narrative review. Cureus. (2018) 10:e3681. doi: 10.7759/cureus.3681 30761233 PMC6367114

[74] Harvard Medical School. National Comorbidity Survey (NCS) (2017). Available online at: https://www.hcp.med.harvard.edu/ncs/index.php (Accessed January 11, 2024).

[75] NIH. Any anxiety disorder. Available online at: https://www.nimh.nih.gov/health/statistics/any-anxiety-disorder#:~:text=An%20estimated%2019.1%25%20of%20U.S.,disorder%20in%20the%20past%20year (Accessed January 11, 2024).

[76] SAMHSA. National Survey on Drug Use and Health (NSDUH) Releases (2021). Available online at: https://www.samhsa.gov/data/release/2021-national-survey-drug-use-and-health-nsduh-releases (Accessed January 11, 2024).

[77] Flint-Taylor J Cooper CL. (2017) Team resilience: Shaping up for the challenges ahead. In M. F. Crane (Ed.), Managing for resilience: A practical guide for employee wellbeing and organizational performance (pp. 129–149). Routledge.

[78] Dietz AS Driskell JE Sierra MJ Weaver SJ Driskell T Salas E. Teamwork under stress. In: The Wiley Blackwell Handbook of the Psychology of Team Working and Collaborative Processes (2017). p. 297–315.

[79] Hartwig A Clarke S Johnson S Willis S. Workplace team resilience: A systematic review and conceptual development. Organizational Psychol Rev. (2020) 10:169–200. doi: 10.1177/2041386620919476

[80] Colléony A Clayton S Couvet D Saint Jalme M Prévot AC. Human preferences for species conservation: Animal charisma trumps endangered status. Biol Conserv. (2017) 206:263–9. doi: 10.1016/j.biocon.2016.11.035

[81] Ward PI Mosberger N Kistler C Fischer O. The relationship between popularity and body size in zoo animals. Conserv Biol. (1998) 12:1408–11. doi: 10.1046/j.1523-1739.1998.97402.x

[82] Williams Institute. (2021). Public and Private Sector Employees’ Perceptions of Discrimination Against LGBTQ People. Available at: https://williamsinstitute.law.ucla.edu/publications/employee-perception-discrim/. (Accessed March 20, 2024).

[83] Brown A. 5 key findings about LGBTQ+ Americans. Pew Research Center (2023). Available at: https://www.pewresearch.org/short-reads/2023/06/23/5-key-findings-about-lgbtq-americans/

[84] Matijczak A McDonald SE. LGBTQIA+ populations and Human-Animal Interactions. In: Fine A Mueller M Ng Z Peralta J Beck A, editors. The Handbook on Human Animal Interactions, Interventions and Anthrozoology. New York, NY, USA: Routledge (2023).

[85] McDonald SE Matijczak A Nicotera N Applebaum JW Kremer L Natoli G. “He was like, my ride or die”: Sexual and gender minority emerging adults’ perspectives on living with pets during the transition to adulthood. Emerging Adulthood. (2022) 10:1008–25. doi: 10.1177/21676968211025340

[86] McGowan B. Pride in our communities: The legal intersection of the LGBTQ and environmental justice movements. Vt J Env’t L. (2022) 24:70.

[87] Muraco A Putney J Shiu C Fredriksen-Goldsen KI. Lifesaving in every way: The role of companion animals in the lives of older lesbian, gay, bisexual, and transgender adults age 50 and over. Res Aging. (2018) 40:859–82. doi: 10.1177/0164027517752149 PMC6027597 29357737

[88] Rosenberg S Riggs DW Taylor N Fraser H. ‘Being together really helped’: Australian transgender and non-binary people and their animal companions living through violence and marginalisation. J Sociology. (2020) 56:571–90. doi: 10.1177/1440783319896413

[89] Ng E Lyons ST Schweitzer L eds. Managing the new workforce: International perspectives on the millennial generation. Cheltenham, UK: Edward Elgar Publishing (2012). doi: 10.4337/9780857933010

[90] Williams ND Fish JN. The availability of LGBT-specific mental health and substance abuse treatment in the United States. Health Serv Res. (2020) 55:932–43. doi: 10.1111/1475-6773.13559 PMC7704474 32970327

[91] Owens B Mills S Lewis N Guta A. Work-related stressors and mental health among LGBTQ workers: Results from a cross-sectional survey. PloS One. (2022) 17:e0275771. doi: 10.1371/journal.pone.0275771 36282835 PMC9595555

[92] Bunderson JS Thompson JA. The call of the wild: Zookeepers, callings, and the double-edged sword of deeply meaningful work. Administrative Sci Q. (2009) 54:32–57. doi: 10.2189/asqu.2009.54.1.32

[93] Good S Keeper IA Zoo CM. The future of zookeeping and the challenges ahead. ZOOS’PRINT. (2009) 24:9–14.

[94] Lannoye-Hall C. Individual and institutional barriers in animal care career diversity at zoos and aquariums. Texas Tech University, Lubbock, TX (2023).

[95] Murphy B Maynard L. Connecting workplace attachment and pro-environmental behaviors in zoo and aquarium professionals. Zoo Biol. (2022) 41:439–47. doi: 10.1002/zoo.21703 35608009

[96] Rende K Fromson K Jones MG Ennes M. The privilege of low pay: Informal educators’ perspectives on workforce equity and diversity. J Museum Educ. (2021) 46:430–40. doi: 10.1080/10598650.2021.1975484

[97] Jenkins JL Jr. Rudd ML. Decolonizing animal welfare through a social justice framework. Front Veterinary Sci. (2022) 8:787555. doi: 10.3389/fvets.2021.787555 PMC8829506 35155644

[98] Areola EMQ Lao RMP Javier RP. Job satisfaction and organizational commitment of millennial and gen z hotel employees: a basis for management technique development. J Sustain Community Dev. (2023) 5:234–76.

[99] Panagioti M Geraghty K Johnson J Zhou A Panagopoulou E Chew-Graham C. Association between physician burnout and patient safety, professionalism, and patient satisfaction: a systematic review and meta-analysis. JAMA Internal Med. (2018) 178:1317–31. doi: 10.1001/jamainternmed.2018.3713 PMC6233757 30193239

[100] Li F Jiang L Yao X Li Y. Job demands, job resources and safety outcomes: The roles of emotional exhaustion and safety compliance. Accident Anal Prev. (2013) 51:243–51. doi: 10.1016/j.aap.2012.11.029 23274477

[101] Brandhorst JK Compton CA. Constructing barriers to Employee Assistance Program use by federal correctional officers. J Appl Communication Res. (2022) 50:497–514. doi: 10.1080/00909882.2022.2032269

[102] Matthews LR Gerald J Jessup GM. Exploring men’s use of mental health support offered by an Australian Employee Assistance Program (EAP): perspectives from a focus-group study with males working in blue- and white-collar industries. Int J Ment Health Syst. (2021) 15:68. doi: 10.1186/s13033-021-00489-5 34348756 PMC8334339

[103] Wu AW Connors CA Norvell M. Adapting RISE: Meeting the needs of healthcare workers during the COVID-19 pandemic. Int Rev Psychiatry. (2021) 33:711–7. doi: 10.1080/09540261.2021.2013783 35412425

[104] APA. Psychologists struggle to meet demand amid mental health crisis (2022). Available online at: https://www.apa.org/pubs/reports/practitioner/2022-covid-psychologist-workload#:~:text=Increased%20workload%20and%20longer%20waitlists&text=The%20percentage%20of%20psychologists%20seeing,down%20from%2065%25%20in%202021 (Accessed January 11, 2024).

[105] Crisan C Van Dijk PA Oxley J De Silva A. Worker and manager perceptions of the utility of work-related mental health literacy programmes delivered by community organisations: a qualitative study based on the theory of planned behaviour. BMJ Open. (2022) 12:e056472. doi: 10.1136/bmjopen-2021-056472 PMC8961141 35351719

[106] Beyer C. Building Mental Health Literacy. The Voice (2022).

[107] Morgan AJ Ross A Reavley NJ. Systematic review and meta-analysis of Mental Health First Aid training: Effects on knowledge, stigma, and helping behaviour. PloS One. (2018) 13:e0197102. doi: 10.1371/journal.pone.0197102 29851974 PMC5979014

[108] Gilbert S Mullen J Kelloway EK Dimoff J Teed M McPhee T. The CARE model of employee bereavement support. J Occup Health Psychol. (2021) 26:405. doi: 10.1037/ocp0000287 34472904

[109] Habarth J Bussolari C Gomez R Carmack BJ Ronen R Field NP. Continuing bonds and psychosocial functioning in a recently bereaved pet loss sample. Anthrozoös. (2017) 30:651–70. doi: 10.1080/08927936.2017.1370242

[110] Packman W Field NP Carmack BJ Ronen R. Continuing bonds and psychosocial adjustment in pet loss. J Loss Trauma. (2011) 16:341–57. doi: 10.1080/15325024.2011.572046

[111] Kogan LR Packman W Bussolari C Currin-McCulloch J Erdman P. Pet death and owners’ Memorialization choices. Illness Crisis Loss. (2022), 10541373221143046.

[112] Fisk GM. The complexity and embededness of grief at work: A social-ecological model. Hum Resource Manage Rev. (2022) 33:100929. doi: 10.1016/j.hrmr.2022.100929

[113] Global Aspect Human Capital Advisors. Barriers to Mobility: A Snapshot.

